# Supplementary material for: Metal chalcogenide hollow polar bipyramid prisms as efficient sulfur hosts for Na-S batteries
Source: Nat Commun. 2020 Oct 16;11:5242. doi: 10.1038/s41467-020-19078-0 (PMC7568557; doi:10.1038/s41467-020-19078-0)
Supplement: Supplementary file 1 — Supplementary Information [file 41467_2020_19078_MOESM1_ESM.pdf]

Supplementary Information

Metal chalcogenide hollow polar bipyramid prisms as efficient sulfur hosts for Na-S batteries

Aslam et al.

Supplementary Note 1. DFT calculation details

Density functional theory (DFT) calculations were performed to understand the interactions of NaPSs with CoX_2 cathode hosts. Spin-unrestricted DFT calculations were performed in the VASP code using projector augmented wave (PAW) pseudopotentials.[1, 2] The SCAN meta-GGA functional has been shown to give accurate structures and energetics for a range of diversely bonded solid and molecular systems.[3, 4] In this study, we use the recently developed SCAN+rvv10 [5, 6] extension to the original SCAN functional, which provides an accurate description of short, intermediate and long-range van der Waals interactions which are known to play an important role in binding in transition metal sulfide materials.[7] The addition of an empirical Hubbard U correction (DFT+U) is commonly used in the literature to improve the description of electron correlation in transition metal oxide systems, however it has previously been shown that standard local spin density approximation (LSDA) calculations result in a better description of the magnetic properties in comparison with experiment than LSDA+U calculations.[8] Unlike in oxide systems,[9] there is also limited data about suitable U values for selenide and telluride systems. The SCAN+rvv10 meta-GGA functional was therefore adopted in this work as opposed to LSDA+U or GGA+U functionals as it has been shown to give a good description of the electronic structure of highly correlated systems without a strong dependence on the Hubbard U parameter.[10]

Bulk phases of CoS_2 and $CoTe_2$ were modelled with the pyrite (*Pa*-3) and marcasite (*Pnnm*) structures, respectively, and will subsequently be referred to as p-CoS_2 and m-$CoTe_2$. Several functionals were tested for the p-CoS_2 structure and SCAN+rvv10 was found to give the closest agreement with the experimental lattice parameters, as shown in **Supplementary Figure 23**.

The unit cells and atomic positions of all bulk phases were optimized until the force on any atom was less than 0.01 eV, with a total energy convergence criterion of 10^{-6} eV. A gamma-centred k-point mesh with a mesh density of 36 Å or higher was used to sample the Brillouin zone for all bulk structures. A plane-wave basis set cut-off energy of 400 eV was used unless otherwise stated.

The surface energies of the p-CoS_2, m-$CoSe_2$ and m-$CoTe_2$ structures were calculated with slab calculations. The slab calculations for p-CoS_2, m-$CoSe_2$ and m-$CoTe_2$ were performed using the PBEsol functional for different surfaces from bulk relaxed geometries. All slabs were generated using the Python Materials Genomics (pymatgen) package and only non-polar surfaces were considered in this study.[11] First, a single point calculation was performed for all the slabs from which rough surface energies were calculated. Then, the 8 lowest energy surfaces for p-CoS_2 and the 10 lowest energy surfaces each for m-$CoSe_2$ and m-$CoTe_2$ were fully relaxed to find the most stable surface. The atomic positions for a given surface were optimized (using the input parameters described above) by making a supercell of the slab with 4 layers where the bottom 2 layers were frozen to represent the bulk geometry. A vacuum gap of at least 15 Å was maintained to minimize interactions between periodic images along the z-direction.

The energetics of Na_2S_y polysulfide (y=1, 2, 4, 5 and 6) molecules were modelled in the gas phase in a cubic box with cell lengths of 20 Å, with a single k-point at the gamma-point. To account for the effects of solvation of the Na_2S_y molecules by the liquid TEGDME solvent, an implicit solvation model was included using the VASPsol package.[12] A dielectric constant of $=$ 7.63 was chosen to approximate the dielectric constant of a glyme electrolyte at room

temperature. The atomic positions of all atoms were relaxed in the presence of the implicit solvent.[13] The energetics of solid Na_2S (*Fm*-3*m*), -Na_2S_2 (*P*6_3/*mmc*), Na_2S_4 (*I*-42*d*), Na_2S_5 (*Pnma*), S (*Fddd*) and Na metal (*Im*-3*m*) were also modelled for reference.

The binding energies of Na_2S_y polysulfides to the CoX_2 surfaces were calculated using supercells of the low energy slabs found with PBEsol. For all slabs, the lattice parameters were fixed to the bulk SCAN+rvv10 lattice parameters in the *x* and *y* directions, with at least 15 Å of vacuum between slabs and a plane wave cut-off of 400 eV. Tests were performed with a larger vacuum thickness of ~30 Å for the binding of the Na_2S_4 molecule on the CoS_2 (100) surface and it was found that the binding energies varied by less than 5 % relative to the 15 Å vacuum spacing. A vacuum thickness of ~15 Å was therefore chosen for all subsequent calculations as a balance between accuracy and computational efficiency. A gamma centered k-point mesh with a mesh density of 22 Å or better was used in the *x* and *y* directions, with a single k-point along the z-direction. The bottom two layers of the slabs were fixed, and the top two layers were allowed to fully relax. Geometry optimizations were initialized for each Na_2S_y polysulfide from several different starting configurations. For all binding calculations, an implicit solvent was applied with the VASPsol package as described previously. For comparison, the binding of Na_2S_y polysulfides to a single sheet of graphene were calculated, in a supercell containing 32 C atoms with implicit solvation. A sheet spacing of 32 Å was used along the *z*-direction and larger planewave cut-off of 600 eV was used to give converged results for C. All other parameters were the same as those used for the CoX_2 calculations.

Supplementary Figures

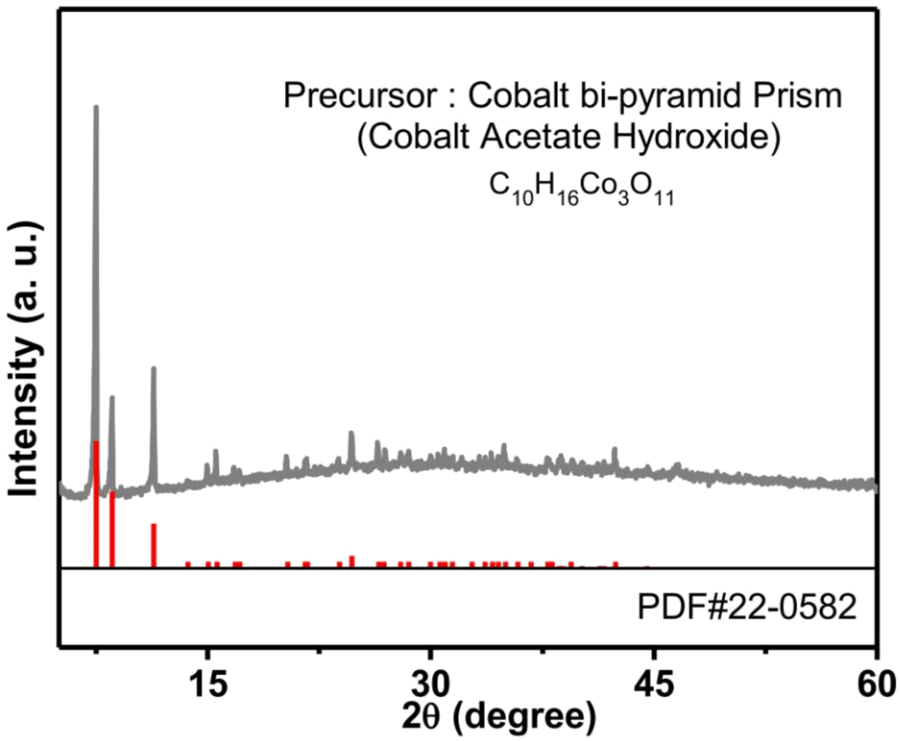

Supplementary Figure 1. XRD pattern of the precursor for the Co-bipyramid prisms.

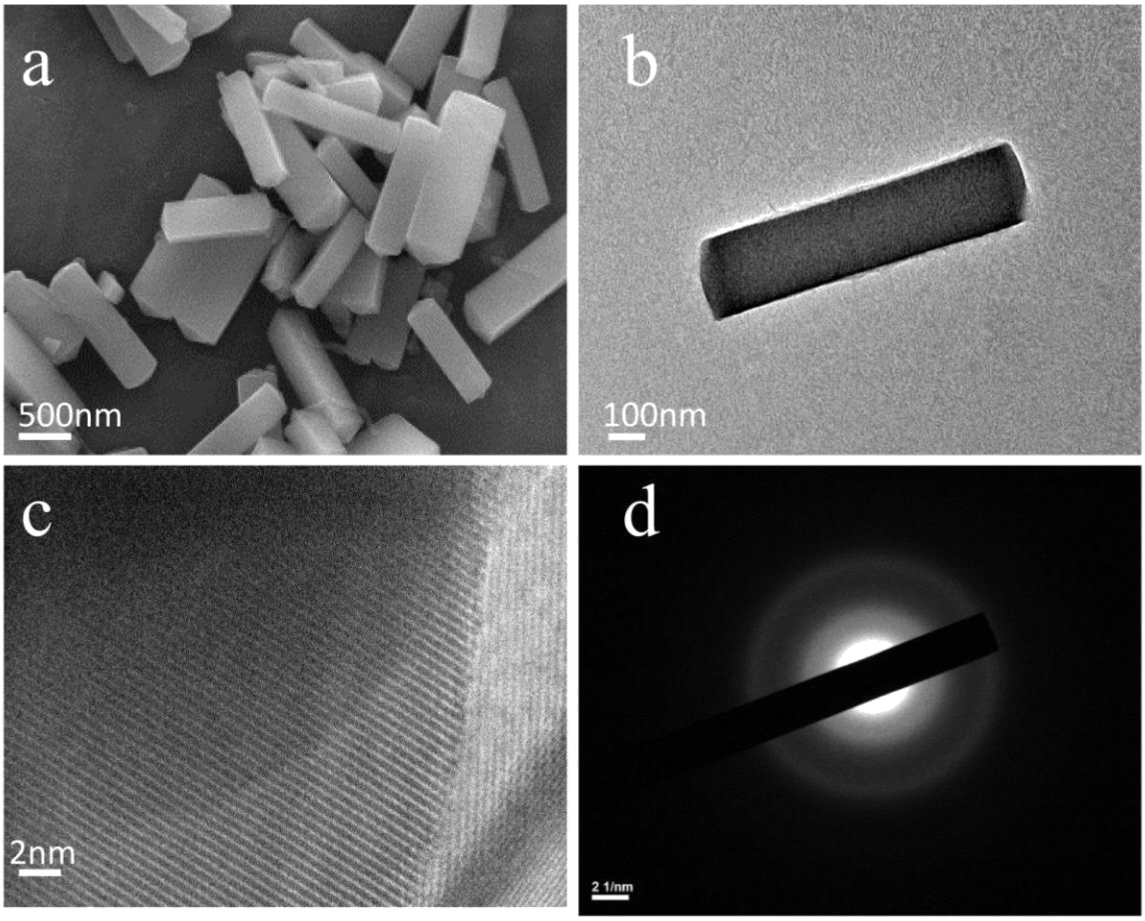

Supplementary Figure 2. **a** FESEM images of the Co-prisms precursor. **b** low resolution TEM of the Co-prisms. **c** high resolution TEM (HRTEM) of the Co-prisms precursor and **d** SAED of Co-prisms precursor.

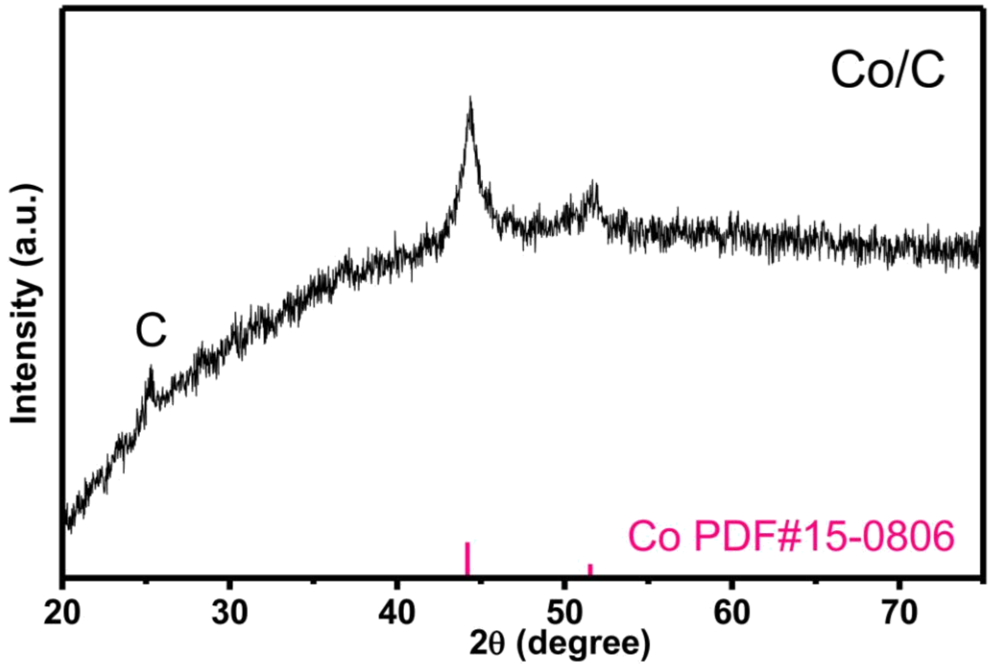

Supplementary Figure 3. XRD pattern of hollow Co/C bipyramid like prisms.

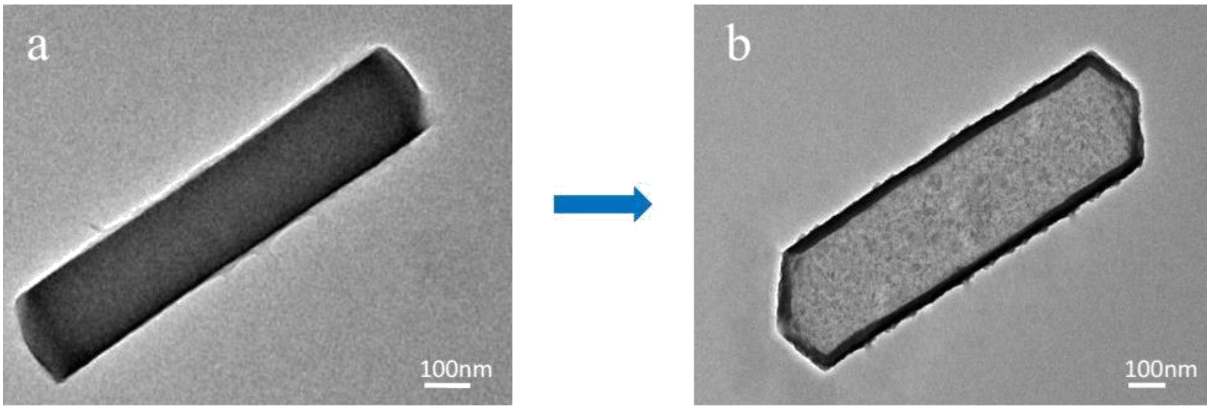

Supplementary Figure 4. **a** TEM images of Co precursor prisms and **b** hollow Co/C.

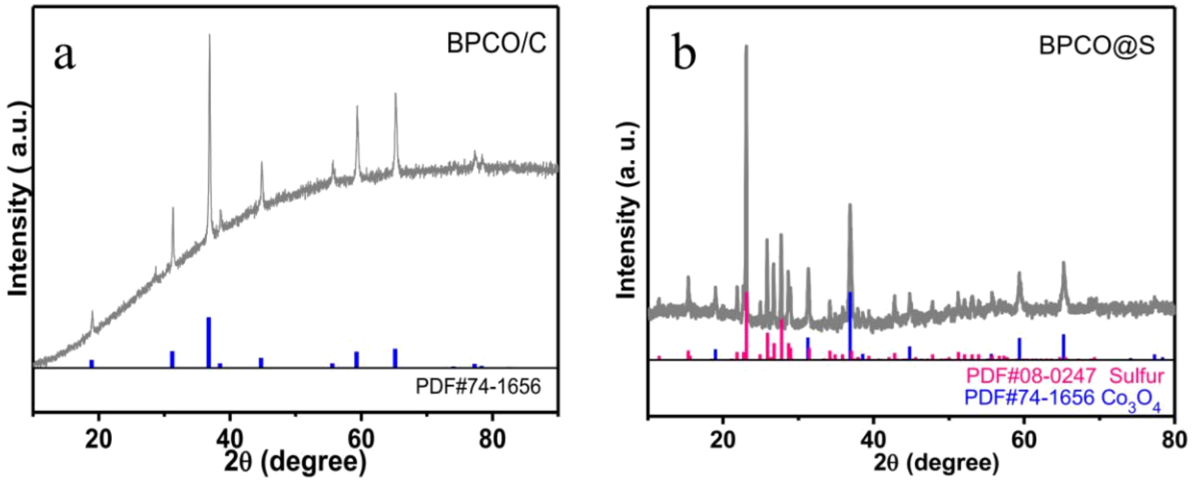

Supplementary Figure 5. **a** XRD patterns of the BPCO and **b** S@BPCO composite.

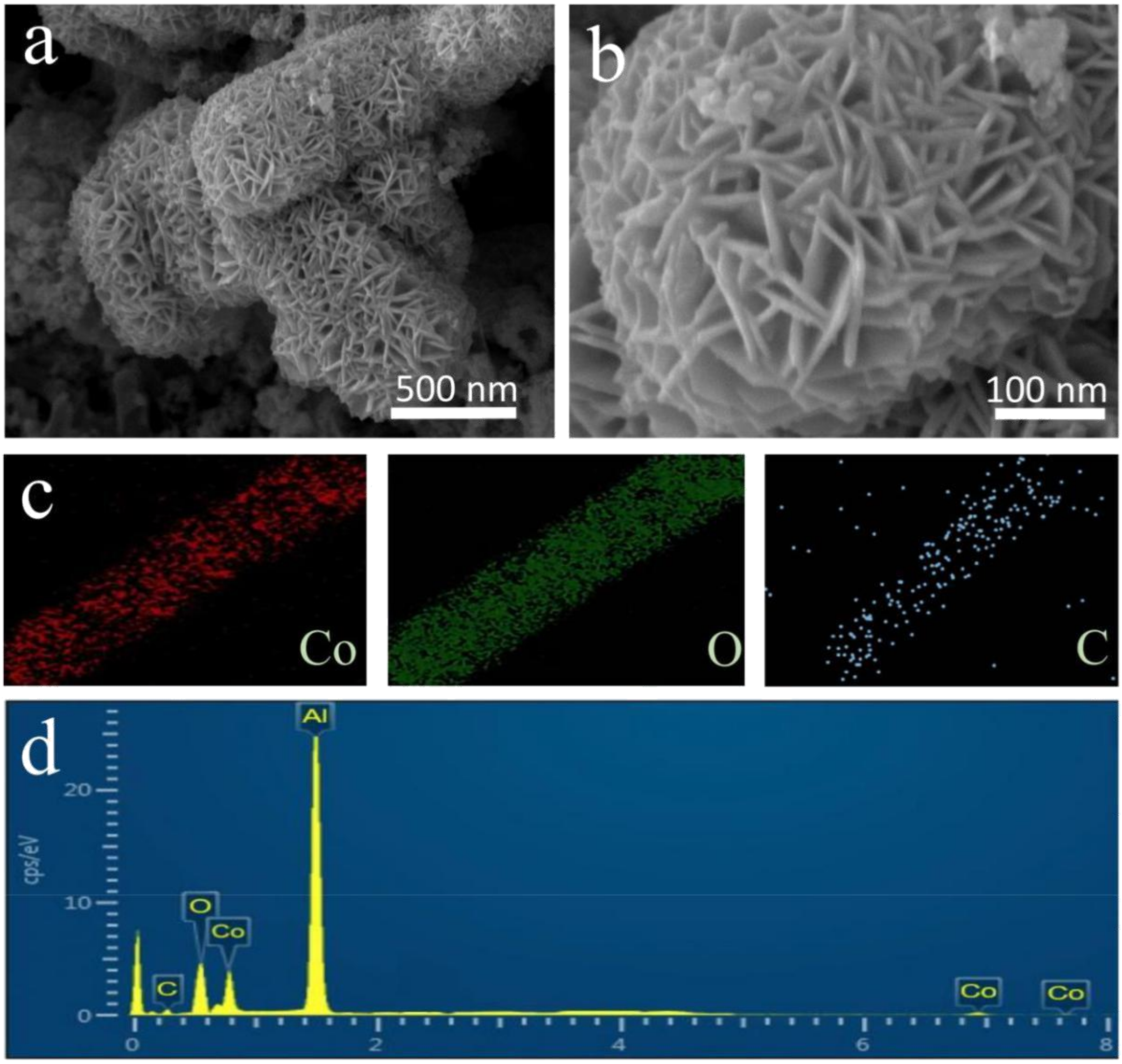

Supplementary Figure 6. **a, b** FESEM images of BPCO. **c** EDS elemental mapping BPCO and **d** EDS spectra.

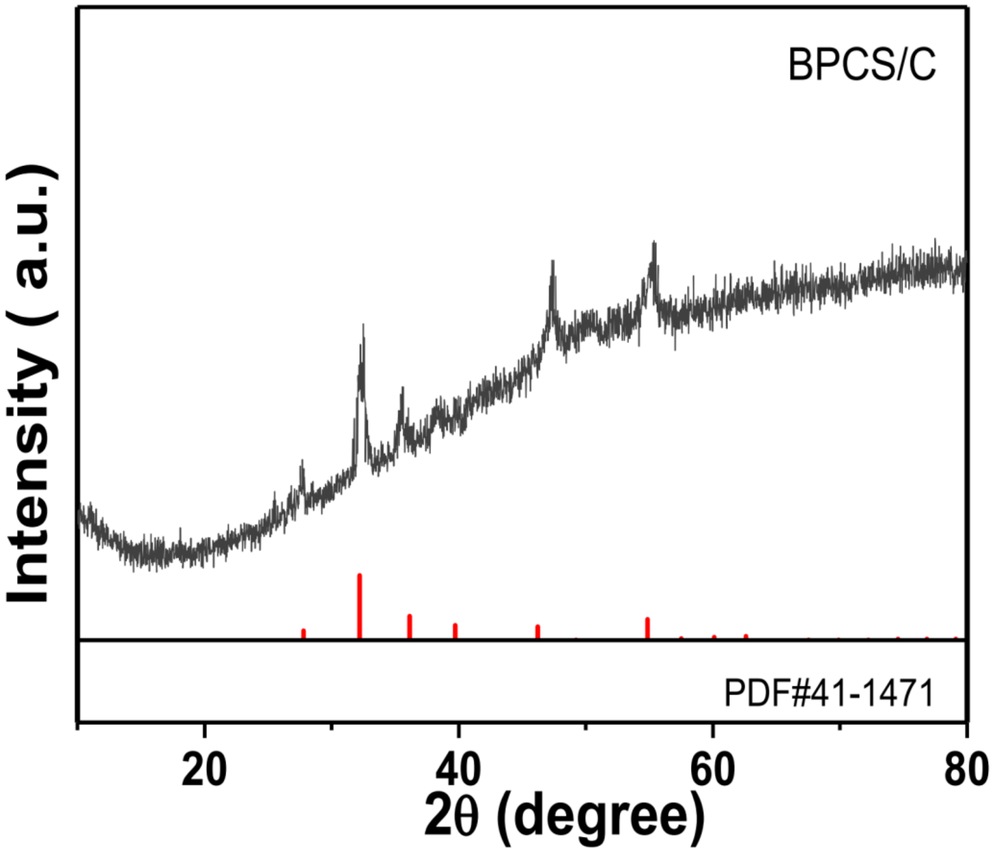

Supplementary Figure 7. XRD pattern of BPCS.

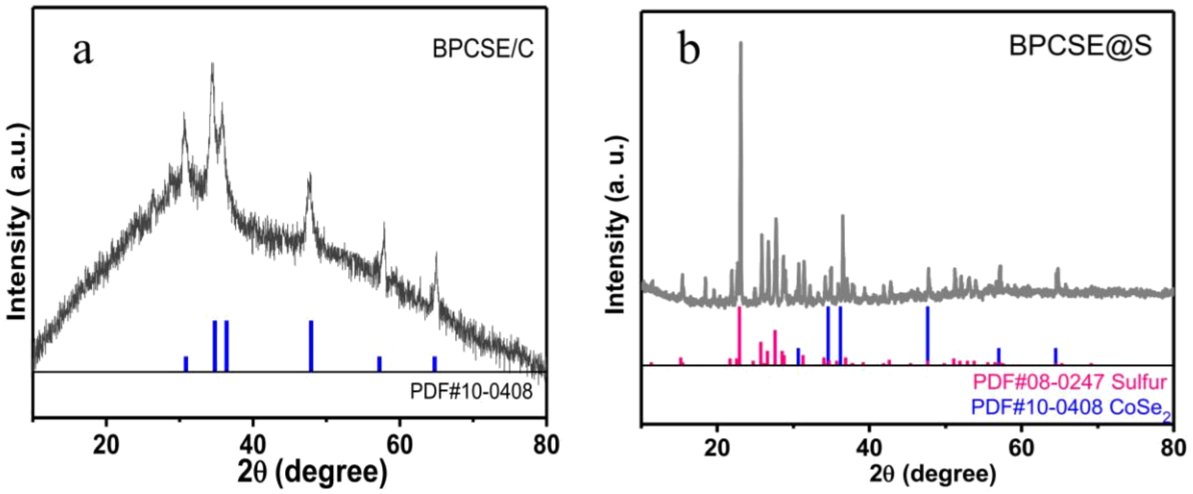

Supplementary Figure 8. **a** XRD pattern of BPCSE and **b** S@BPCSE composite.

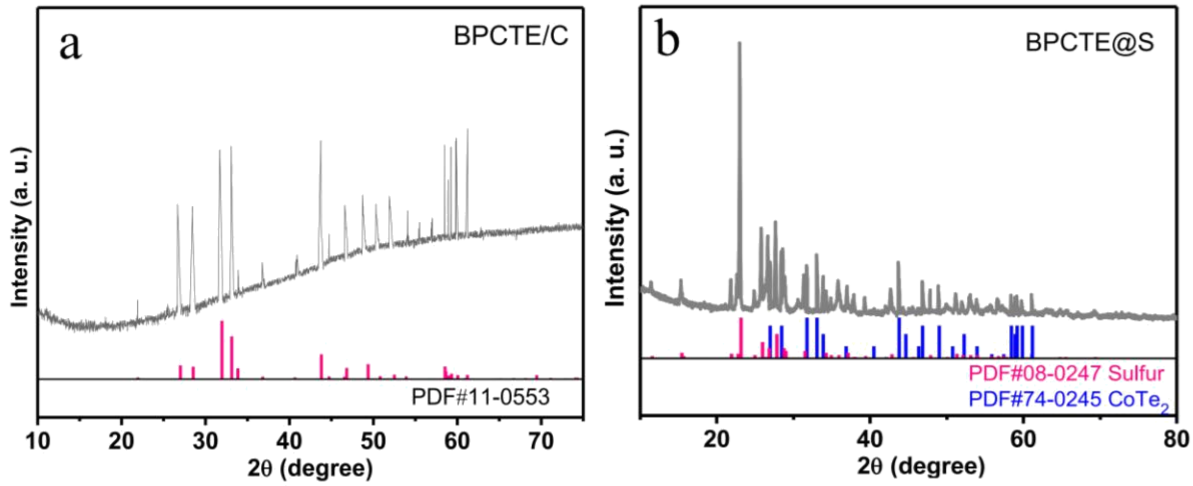

Supplementary Figure 9. **a** XRD pattern of BPCTE and **b** S@BPCTE composite.

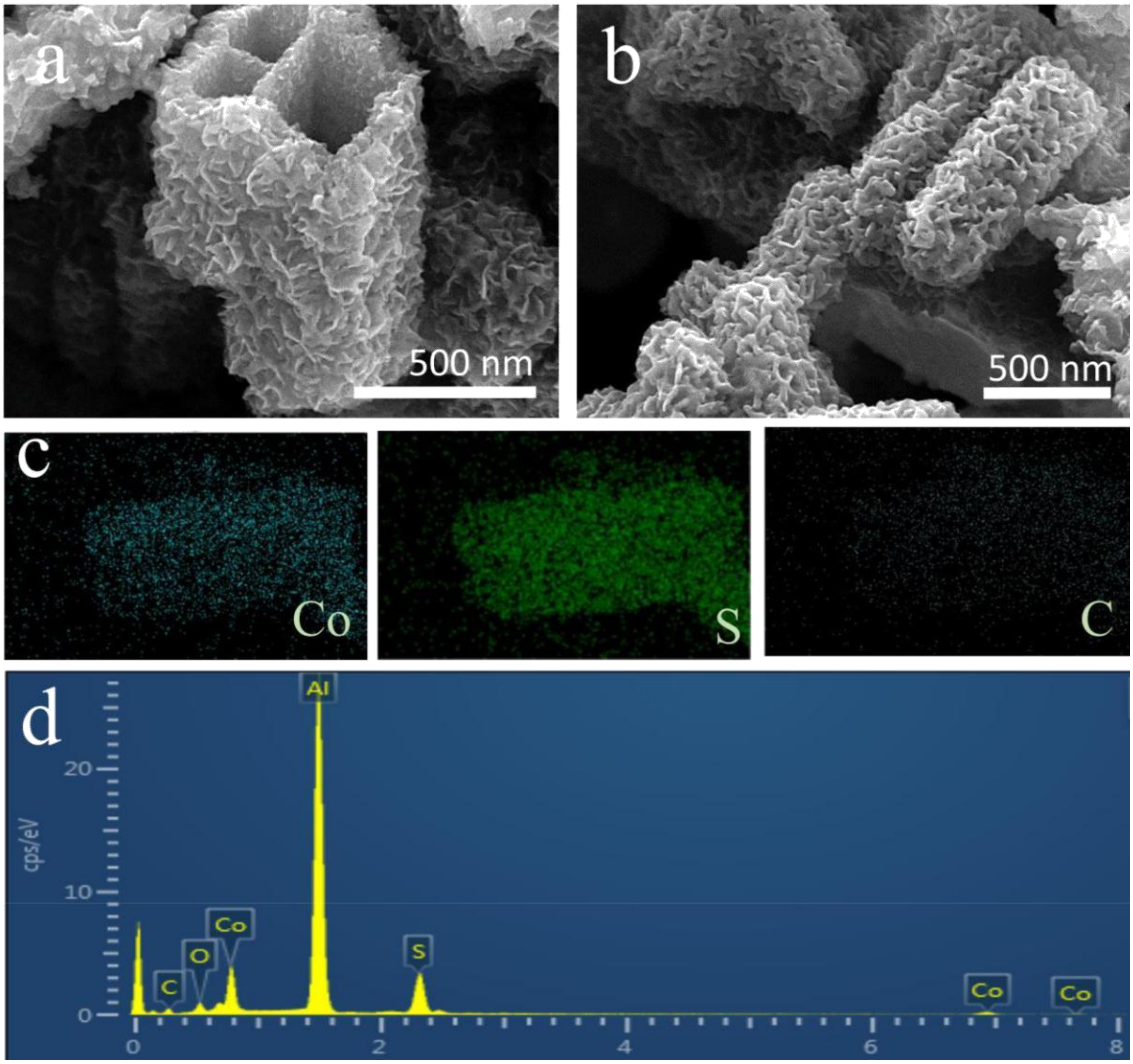

Supplementary Figure 10. **a, b** FESEM images of BPCS. **c** EDS elemental mapping BPCS and **d** EDS spectra.

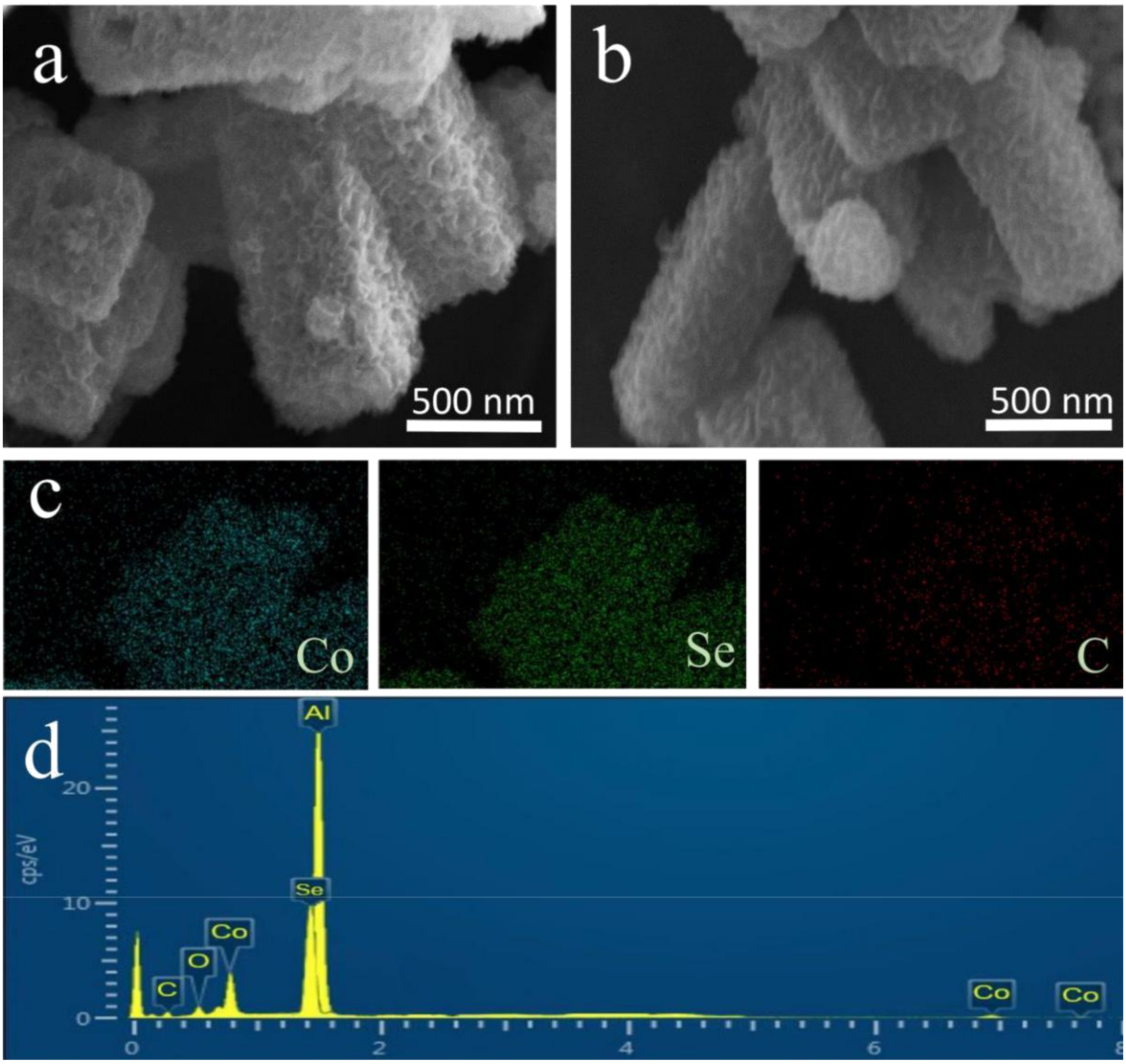

Supplementary Figure 11. **a, b** FESEM images of BPCSE. **c** EDS elemental mapping BPCSE and **d** EDS spectra.

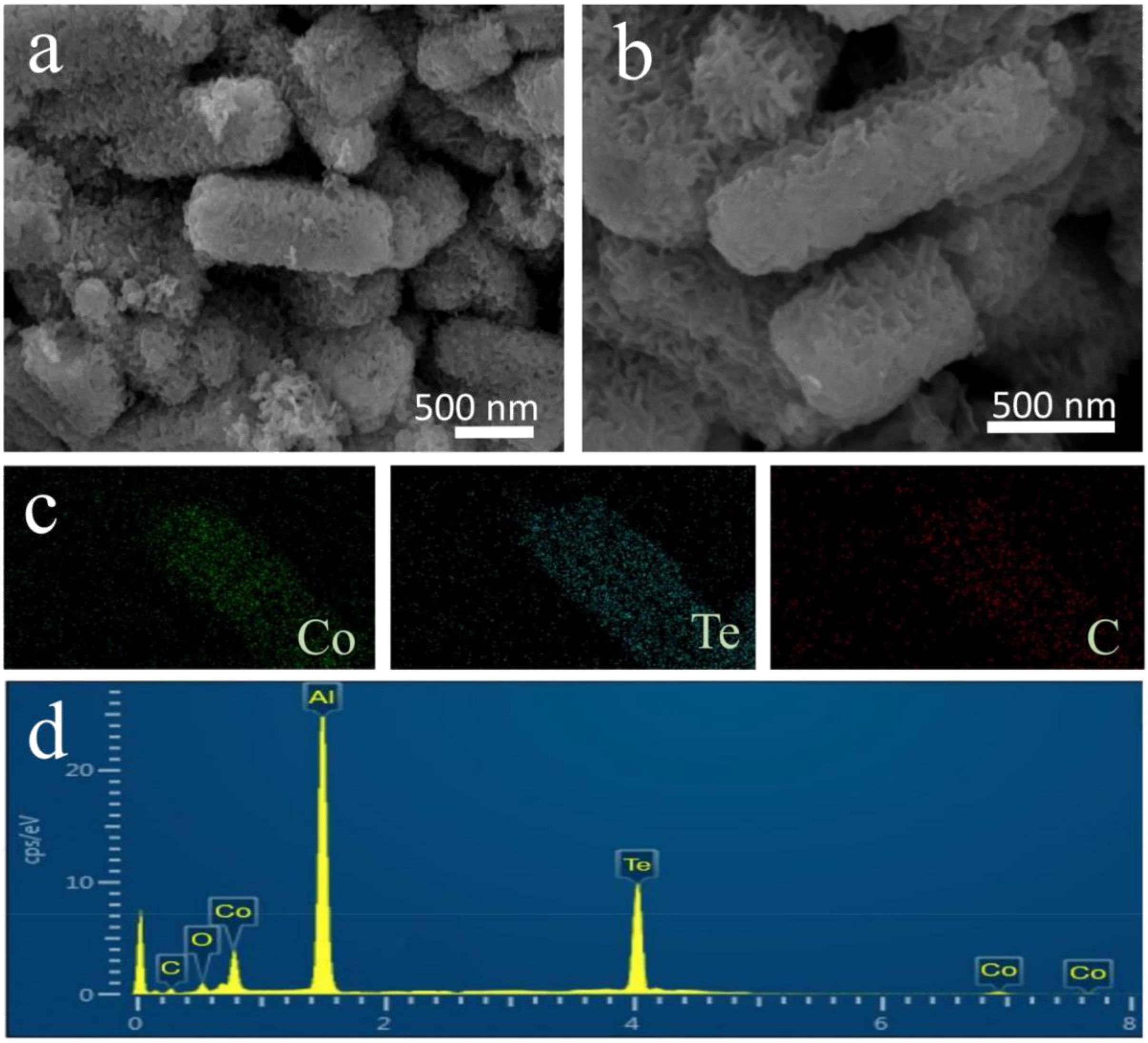

Supplementary Figure 12. **a, b** FESEM images of BPCTE. **c** EDS elemental mapping BPCTE and **d** EDS spectra.

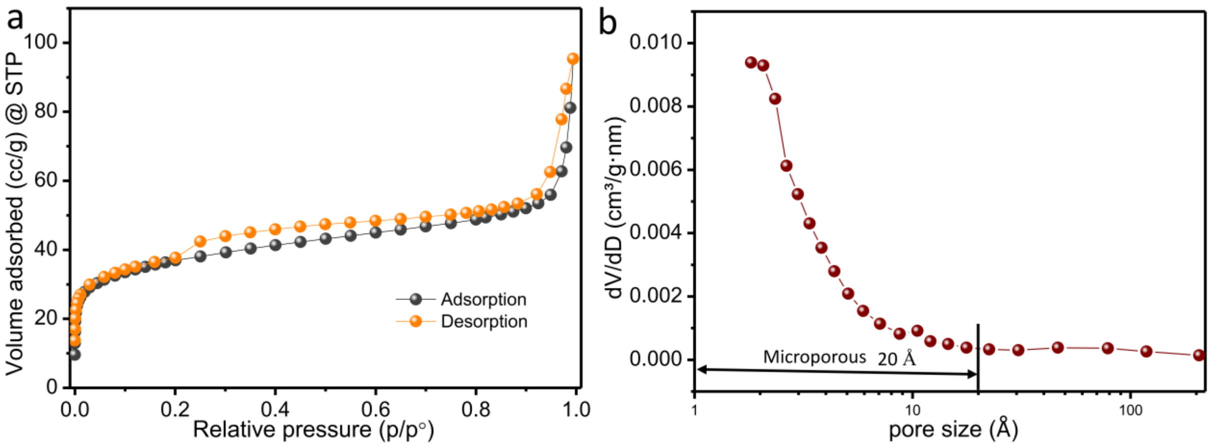

Supplementary Figure 13. **a** N_2 adsorption-desorption hysteresis loop and **b** pore size distribution of BPCS.

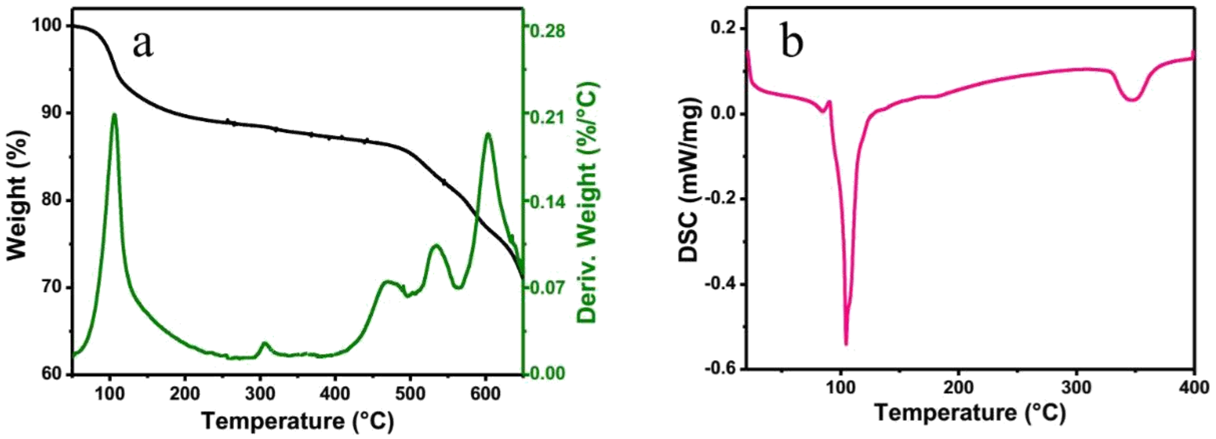

Supplementary Figure 14. **a** TGA curve of pure BPCS bipyramid prisms and **b** DSC curve.

As shown in Figure S9a, BPCS has a mass loss about 13 wt% at 420 °C and the S@BPCS composite has a mass loss about 80 wt% at 420°C, therefore the calculated sulfur loading was 64.5 wt%, (80% - 13%*(1-80%)≈64.5 wt%.

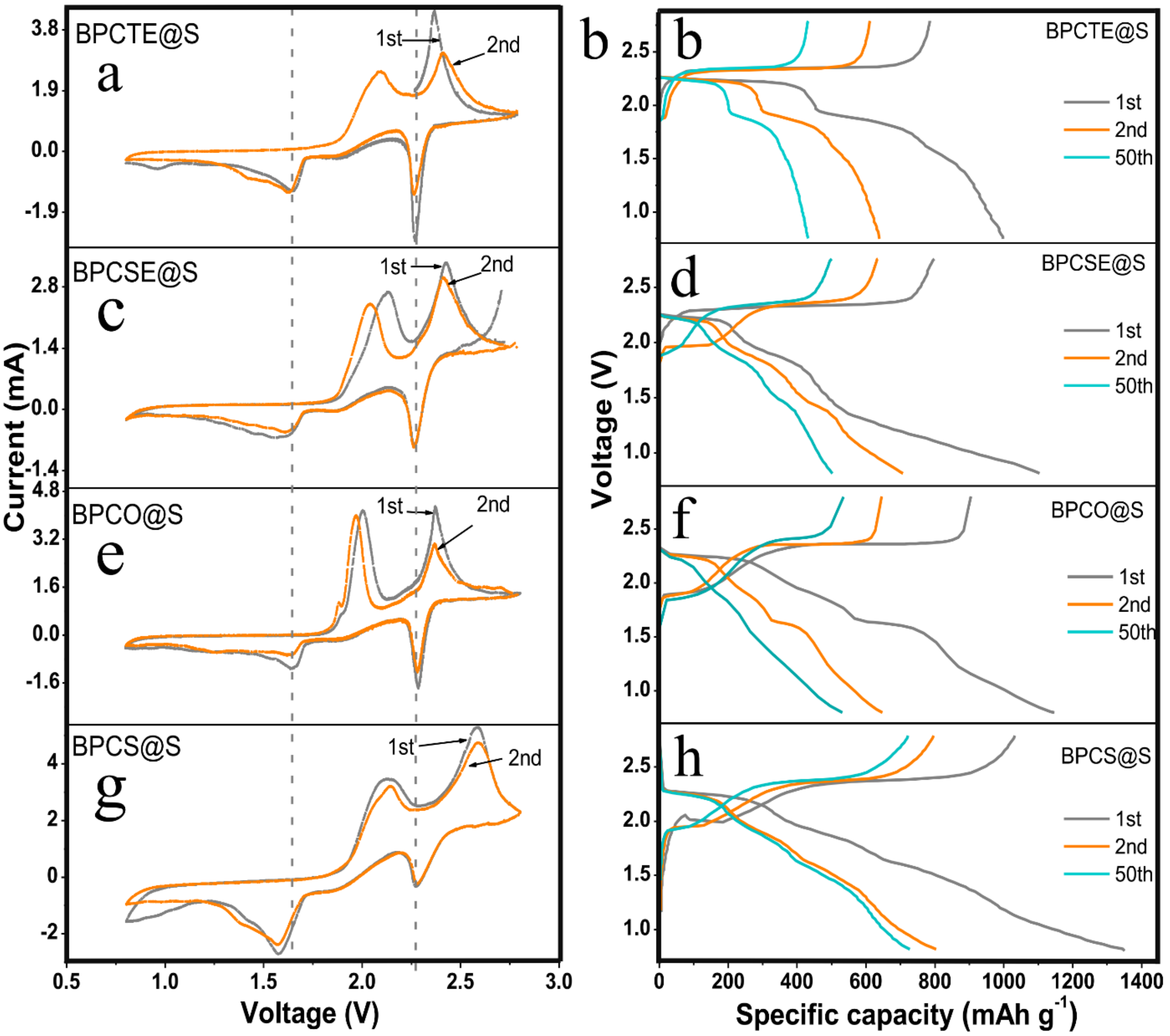

Supplementary Figure 15. CV curves and discharge/charge profiles of the S@chalcogenides. **a, b** BPCTE@S. **c, d** BPCSE@S. **e, f** BPCO@S and **g, h** BPCS@S.

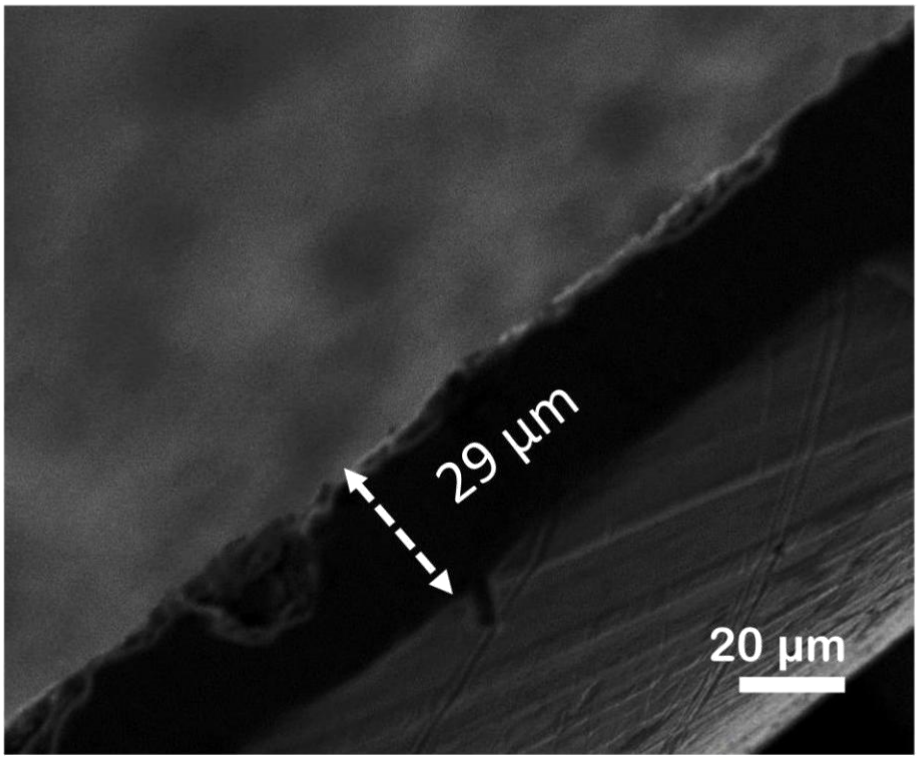

Supplementary Figure 16. SEM image of S@BPCS composite electrode with a mass loading of 4.4 mg cm^{-2}.

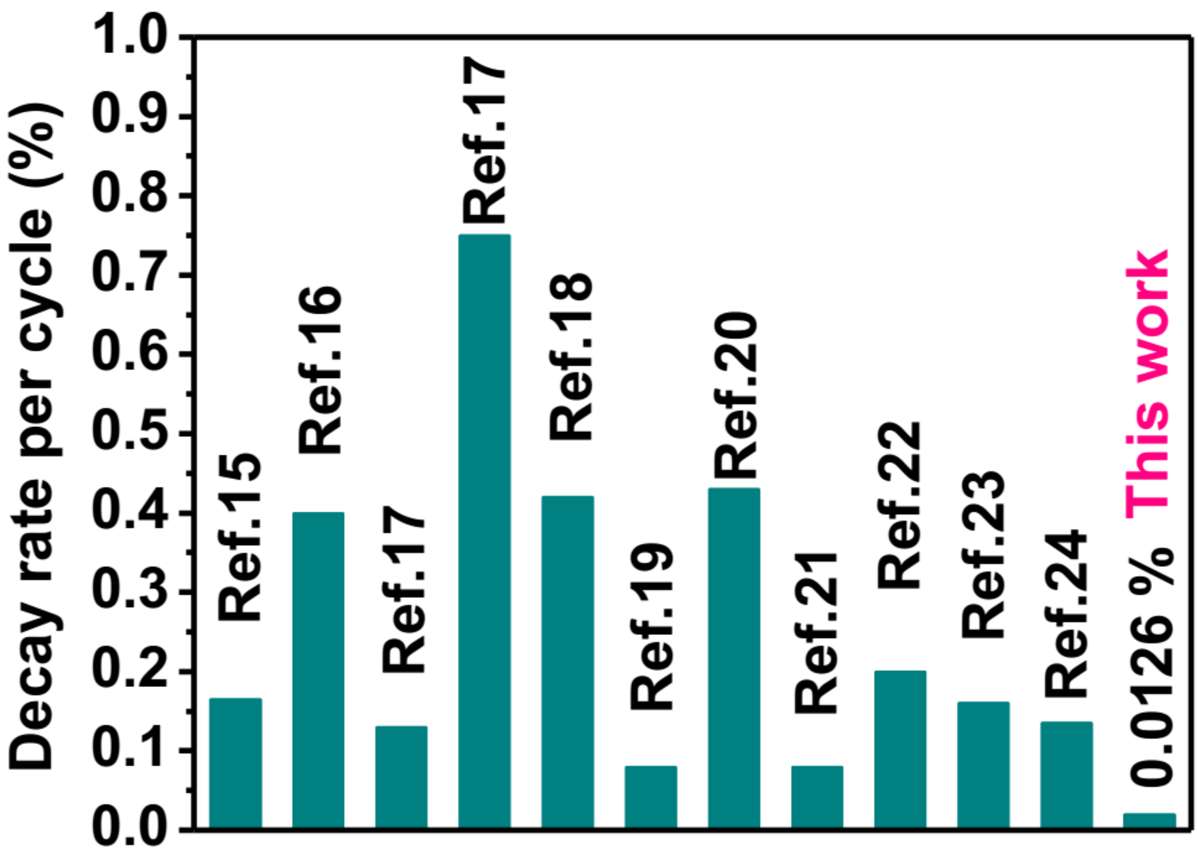

Supplementary Figure 17. Comparison of capacity decay rate of S@BPCS and previously reported Na-S batteries.

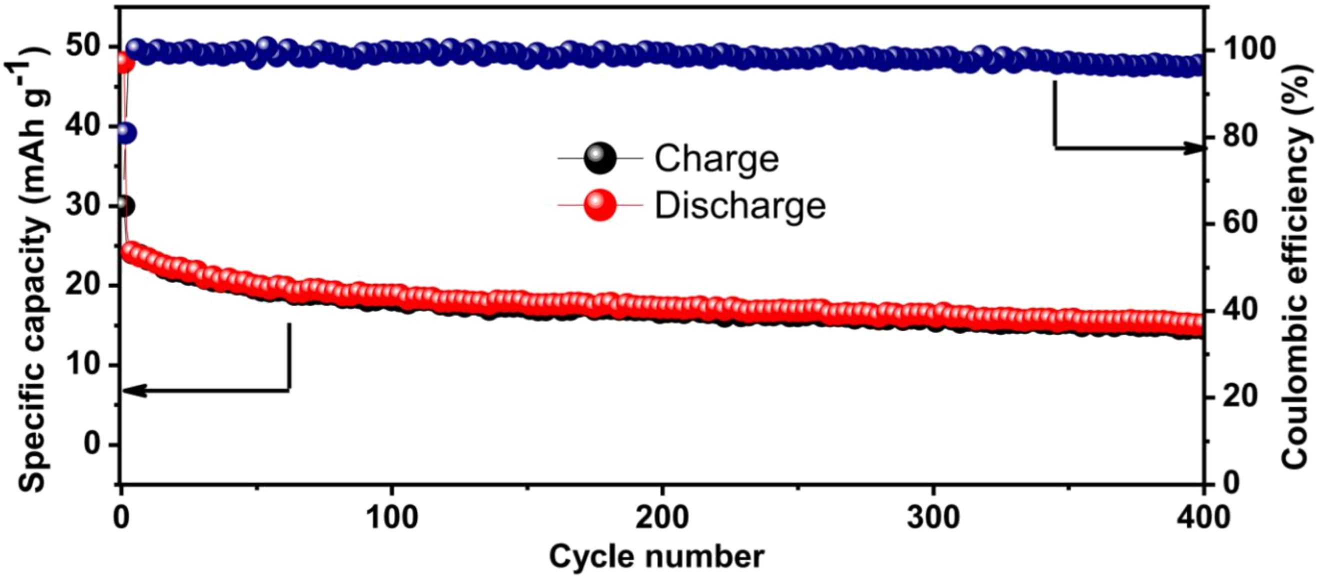

Supplementary Figure 18. Rate performance of BPCS under the same conditions as the S@BPCS composite.

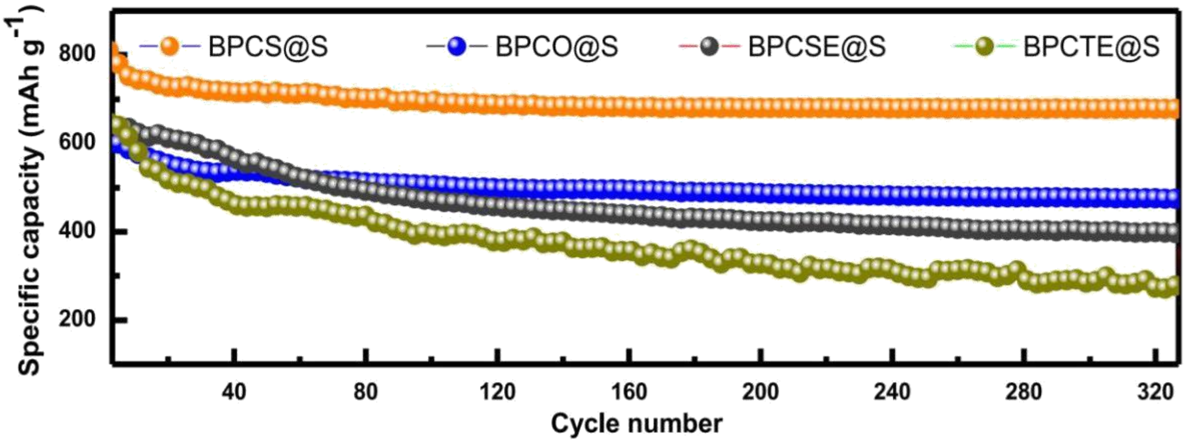

Supplementary Figure 19. Cycling capability of the four composites.

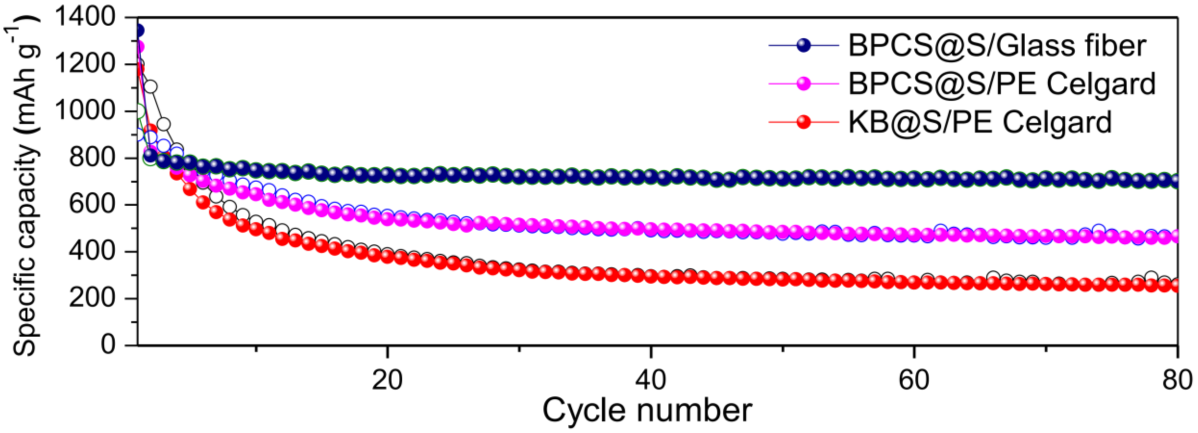

Supplementary Figure 20. Cycling performance of BPCS@S and Ketjen Black@S composites using glass fiber and Celgard polymer as separator.

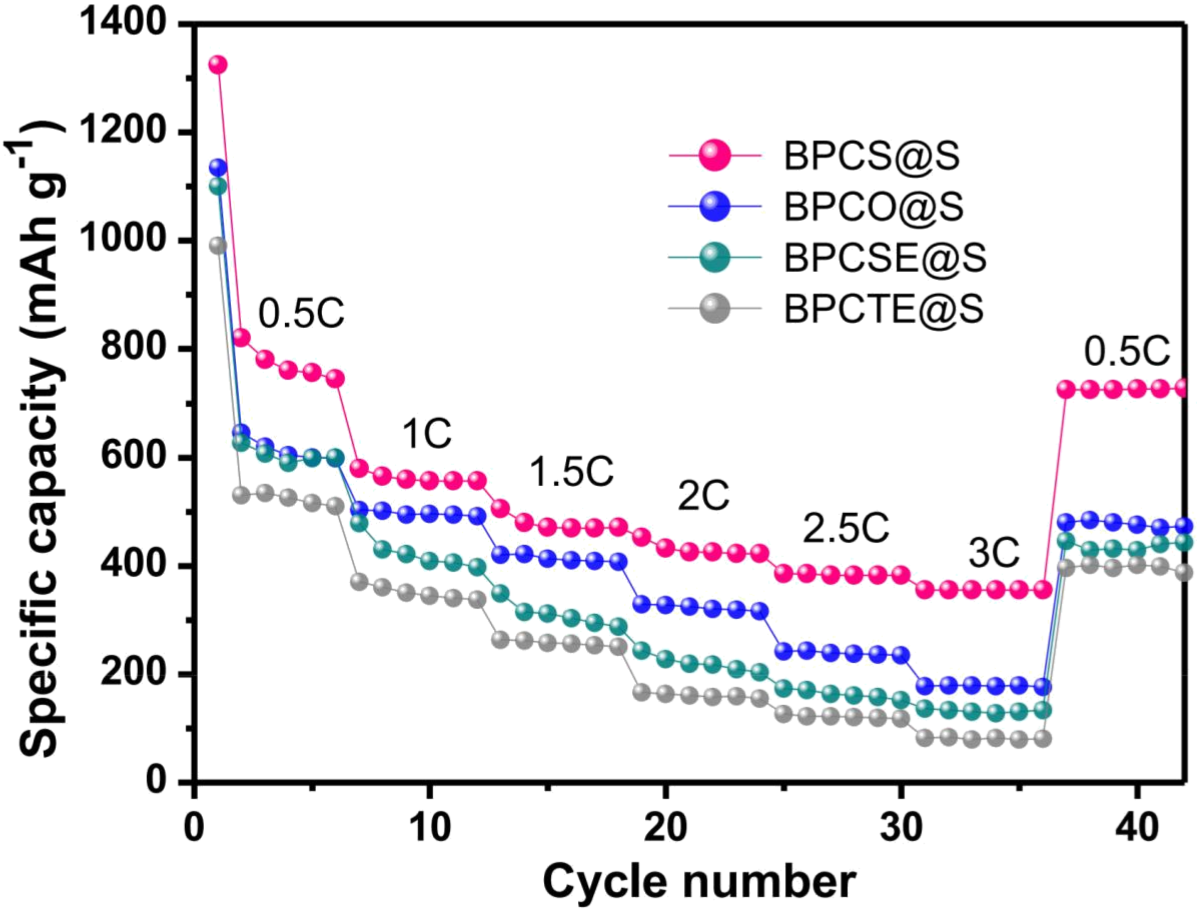

Supplementary Figure 21. Rate capability of the four composites.

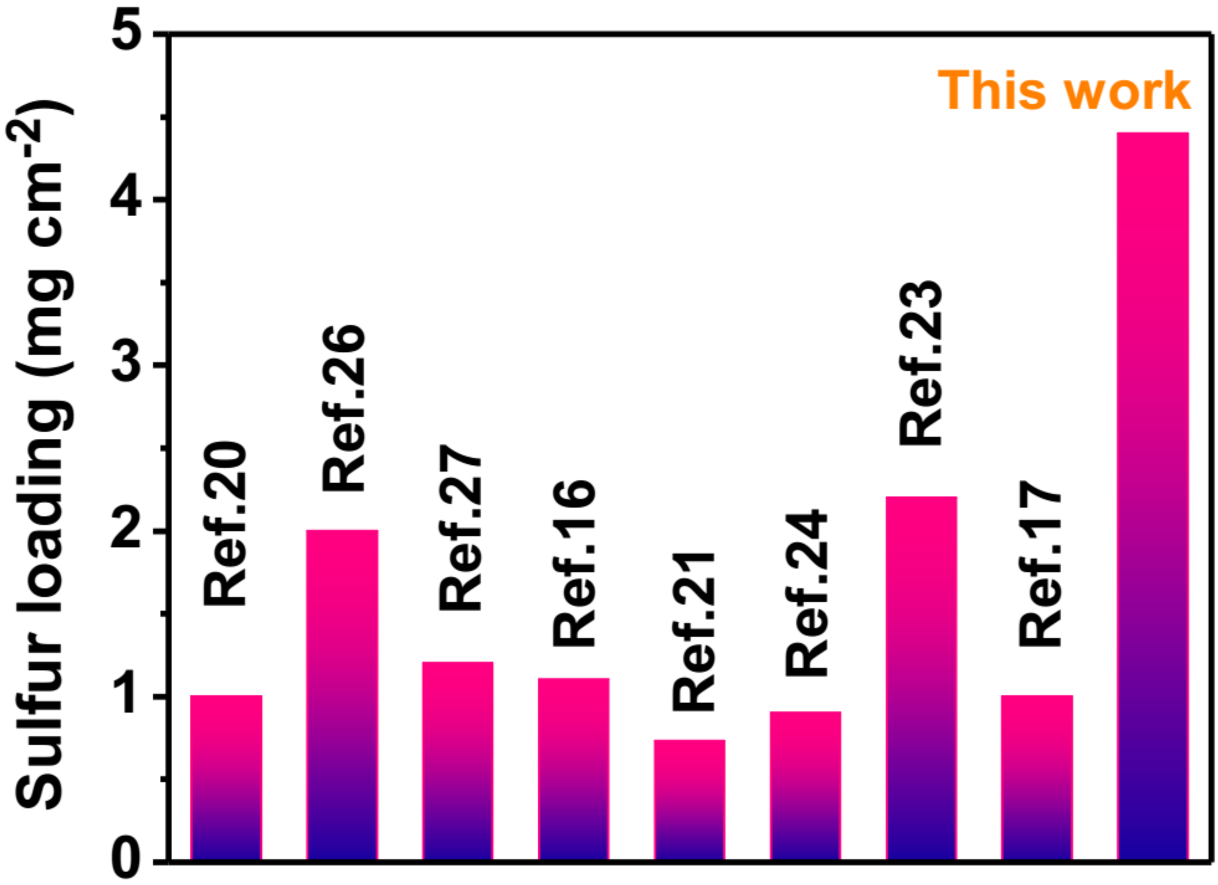

Supplementary Figure 22. Comparison of mass loading in this work and previously reported Na-S batteries.

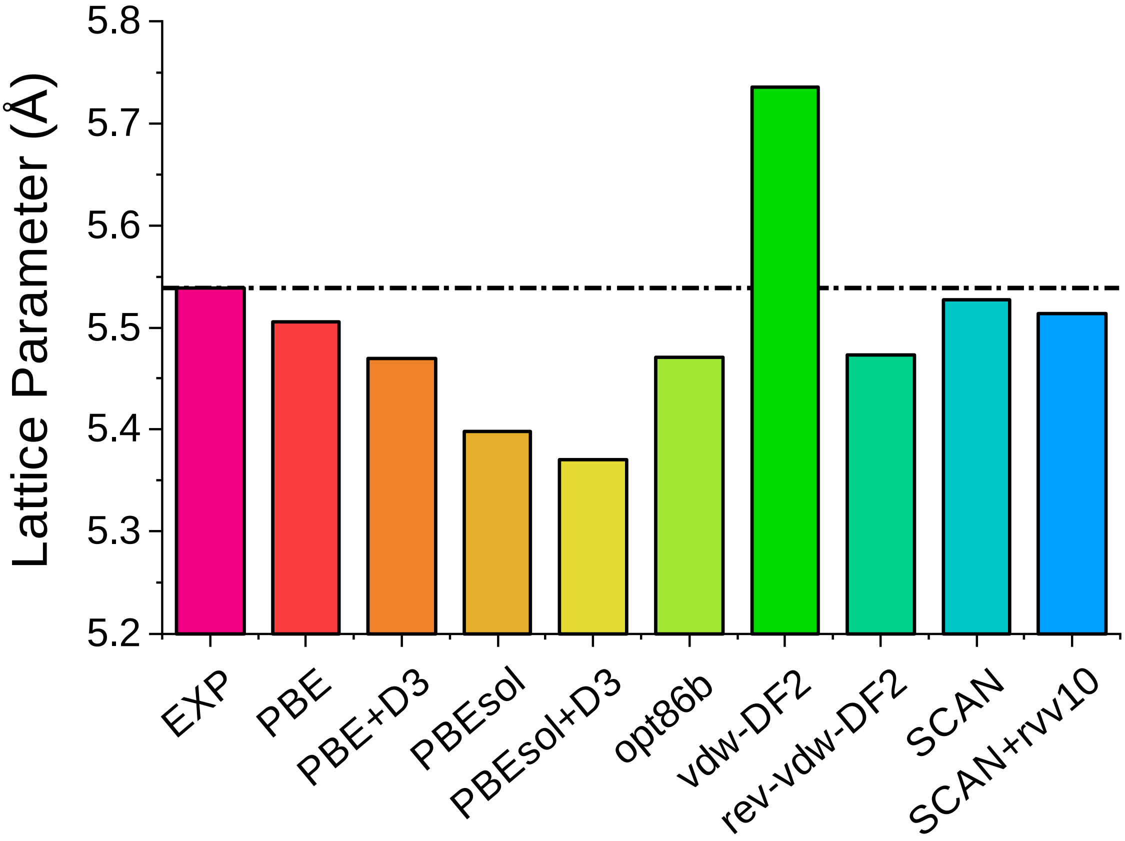

Supplementary Figure 23. Comparison of the lattice parameter of pyrite CoS_2 (Pa-3) optimised with different DFT functionals. The experimental lattice parameter (EXP) is taken from Ref. 14.

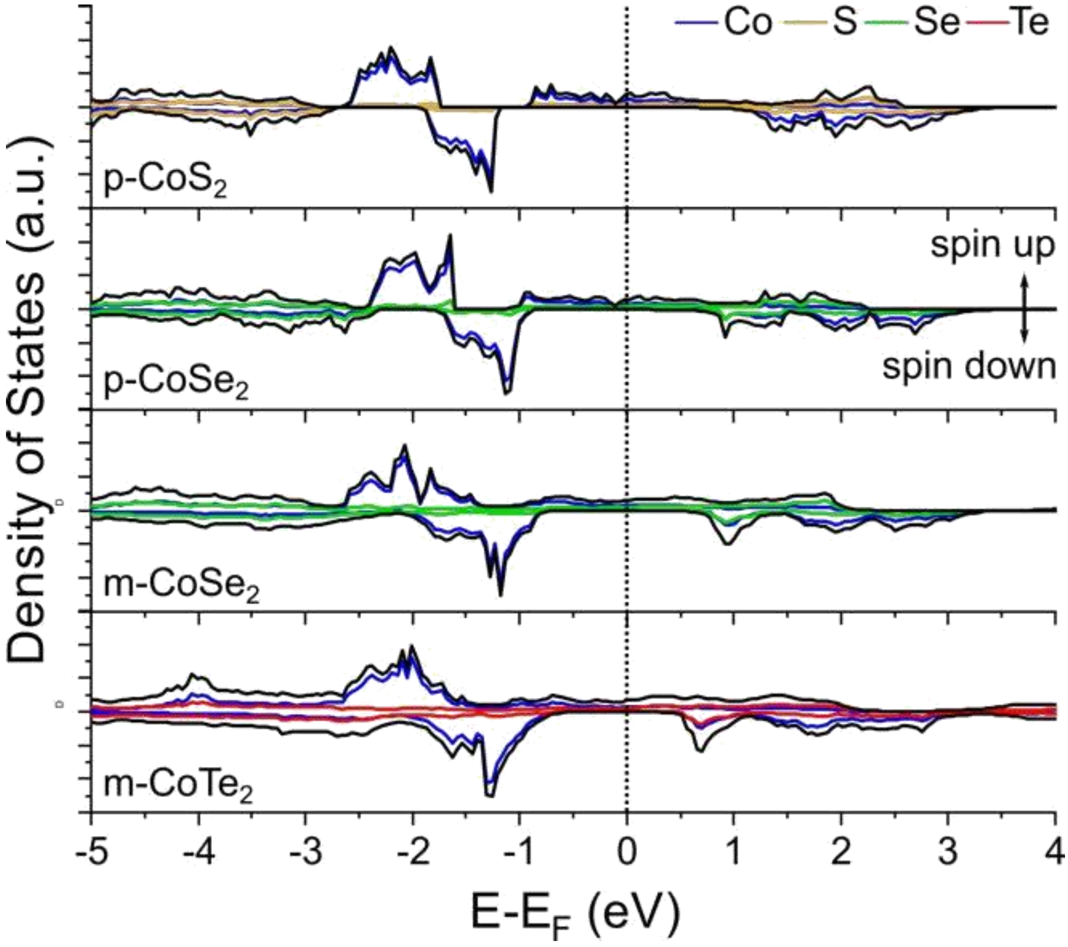

Supplementary Figure 24. Density of states plot of pyrite CoS_2, pyrite $CoSe_2$, marcasite $CoSe_2$ and marcasite $CoTe_2$ computed with SCAN+rvv10 functional. The energy (E) is plotted relative to the Fermi level (E_F) of each structure as shown with a dashed line.

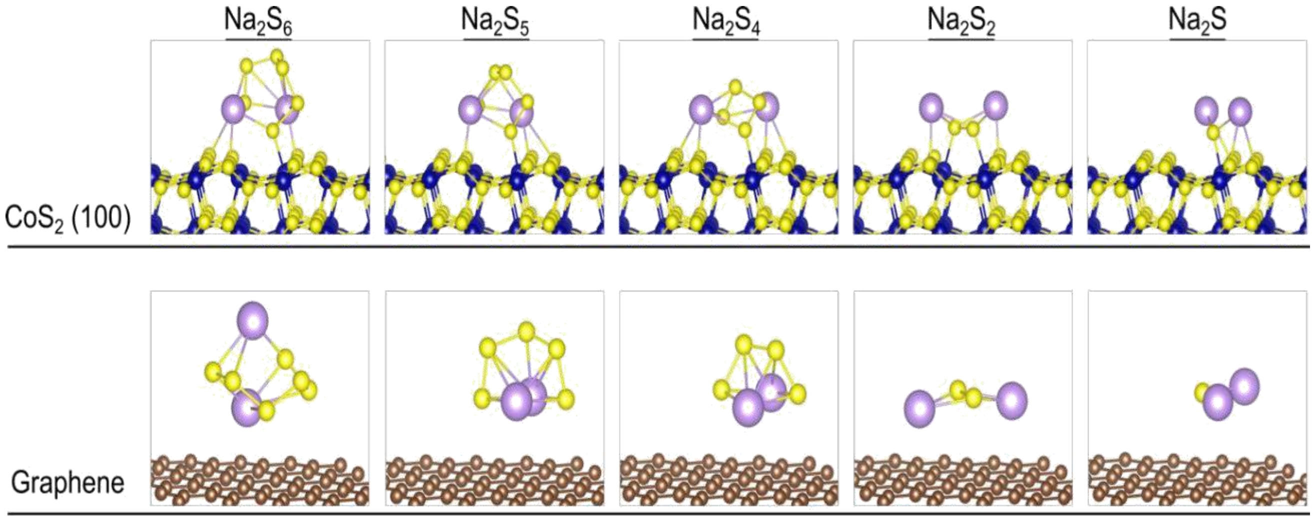

Supplementary Figure 25. Lowest energy configurations of Na_2S_y (y=1, 2, 4, 5 and 6) molecules bound to the pyrite CoS_2 (100) surface and graphene surface in the presence of an implicit solvent. Na, S, Co and C atoms are shown as purple, yellow, blue and brown respectively.

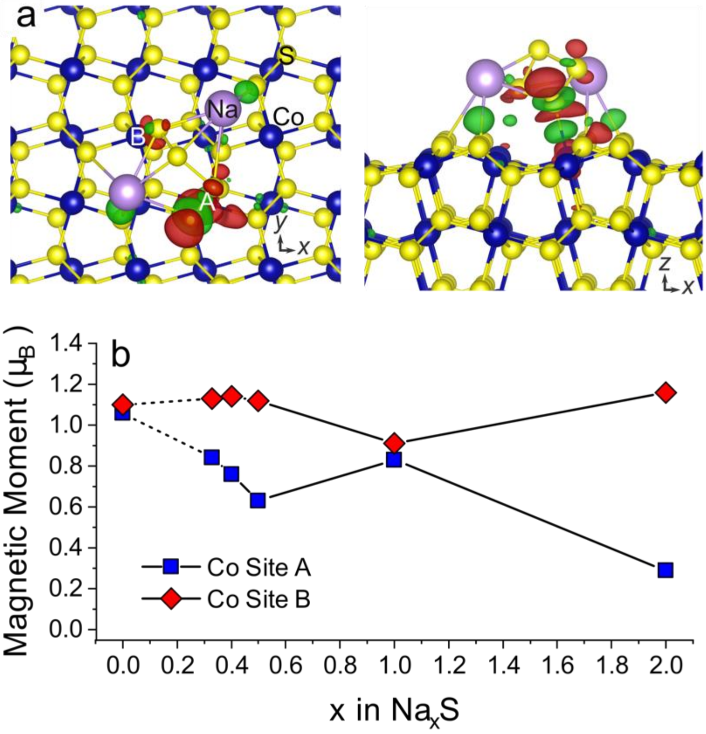

Supplementary Figure 26. (a) Charge difference map (positive: red, negative: green) of Na_2S_4 bound to the p-CoS_2 (100) surface with isosurface level 0.003 e/a_0^3. Two Co^{2+} surface binding sites, A and B, adjacent to the Na_2S_4 adsorbate are labelled. (b) Variation in the magnetic moment of Co site A and Co site B for Na_2S_y adsorbates on the CoS_2 (100) surface. The magnetic moments of the A and B sites without an adsorbate correspond to x=0.

The nature of the binding of Na_2S_y molecules with the CoX_2 surfaces can be visualized from the difference in the charge density before and after binding, as shown for Na_2S_4 bound to the CoS_2 (100) surface **Supplementary 26a**. **Supplementary Figure 26a** shows that charge transfer occurs from the surface S atoms to the Na in the Na_2S_4 cluster, resulting in strong chemical interaction. One of the terminal S atoms in the Na_2S_4 also interacts with a square pyramidal (CoS_5) Co^{2+} site on the surface forming a stable octahedral (CoS_6) environment, which is accompanied by charge transfer from Co to S. The oxidation of the Co^{2+} binding site as a result of charge transfer from all of the Na_2S_y molecules on the CoS_2 (100) surface was probed by analyzing the change in the Co magnetic moment, as shown in **Supplementary Figure 26b.**

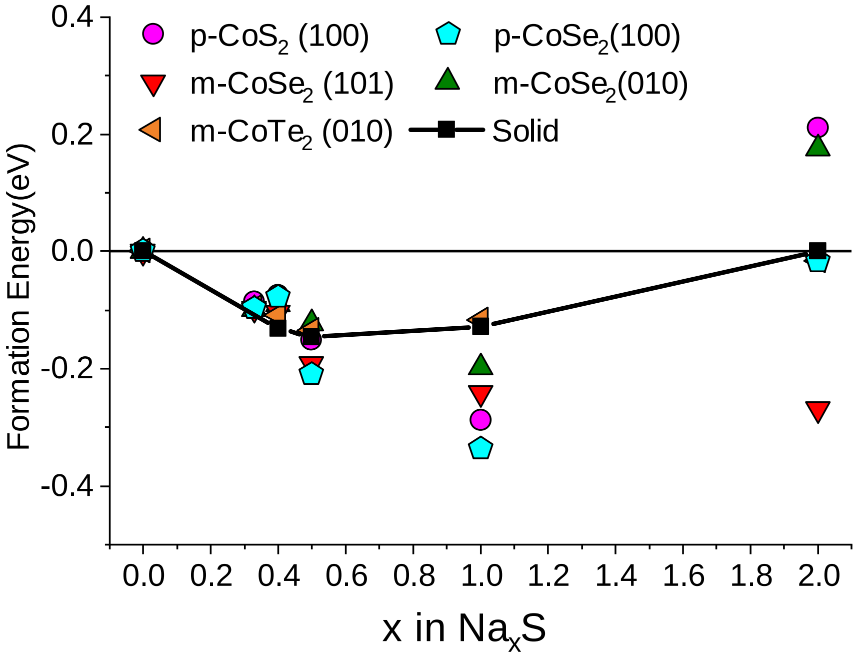

Supplementary Figure 27. Convex energy hull of Na_2S_y molecules bound to the lowest energy surfaces of p-CoS_2, m-$CoSe_2$ and m-$CoTe_2$ from DFT calculations in the presence of implicit solvent. The solid Na_2S_y phase are shown for reference (black line). All energies are normalized per mol of S (x=2/y) and plotted relative to the solid end member phases of Na_2S and S.

For the slab without an adsorbed Na_2S_y molecule, the magnetic moment on all surface Co sites is approximately 1.08 μ_B, which is consistent with the electronic configuration of low spin, Co^{2+} ($t_{2g}^6 e_g*^1$) in an octahedral environment. The magnetic moment of the Co surface site nearest the adsorbed Na_2S_y cluster (Co site A) systematically decreases as the S chain length y decreases (x increases), with the exception of Na_2S_2, suggesting a greater degree of Co oxidation for shorter NaPSs. For the Na_2S_2 system, both S atoms were bound to two separate Co^{2+} atoms (Co sites A and B) instead of a single Co^{2+} site, which led to the oxidation of both sites.

Supplementary Table 1. Comparison of experimental and DFT (SCAN+rvv10) optimized lattice parameters for pyrite CoS_2, pyrite $CoSe_2$, marcasite $CoSe_2$ and marcasite $CoTe_2$ phases.

	Lattice Parameter								
	a			*b*			*c*		
Structure	Exp. (Å)	Calc. (Å)	Diff (%)	Exp. (Å)	Calc. (Å)	Diff (%)	Exp. (Å)	Calc. (Å)	Diff (%)
p-CoS_2	5.539	5.514	-0.45	-	-	-	-	-	-
p-$CoSe_2$	5.859	5.862	0.05	-	-	-	-	-	-
m-$CoSe_2$	4.838	4.825	-0.27	5.713	5.859	2.25	3.589	3.646	1.59
m-$CoTe_2$	5.327	5.272	1.03	6.322	6.316	-0.09	3.900	3.915	0.38

Supplementary Table 2. Surface energies, in J/m^2, for pyrite CoS_2, marcasite $CoSe_2$ and marcasite $CoTe_2$ phases calculated with DFT using the PBESol functional.

Surface	p-CoS_2	m-$CoSe_2$	m-$CoTe_2$
(100)	0.67	1.17	0.91
(010)	---	0.78	0.64
(110)	1.74	1.38	1.11
(101)	--	0.71	0.66
(111)	1.90	0.98	0.89
(210)	1.22	1.27	1.08
(211)	1.29	0.86	0.73
(221)	1.49	---	---
(310)	---	1.27	1.05
(301)	---	1.22	1.07
(311)	1.40	1.07	0.90
(410)	1.42	---	---

Supplementary References

1. Kresse G, Furthmüller J. Efficient iterative schemes for ab initio total-energy calculations using a plane-wave basis set. *Phys. Rev. B* **54**, 11169-11186 (1996).

2. Blöchl PE. Projector augmented-wave method. *Phys. Rev. B* **50**, 17953-17979 (1994).

3. Sun J, Ruzsinszky A, Perdew JP. Strongly Constrained and Appropriately Normed Semilocal Density Functional. *Phys. Rev. Lett.* **115**, 036402 (2015).

4. Sun J, *et al.* Accurate first-principles structures and energies of diversely bonded systems from an efficient density functional. *Nat. Chem.* **8**, 831-836 (2016).

5. Peng H, Yang Z-H, Perdew JP, Sun J. Versatile van der Waals Density Functional Based on a Meta-Generalized Gradient Approximation. *Phys. Rev. X* **6**, 041005 (2016).

6. Sabatini R, Gorni T, De Gironcoli S. Nonlocal van der Waals density functional made simple and efficient. *Phys. Rev. B* **87**, 041108 (2013).

7. Zhang Q, Wang Y, Seh ZW, Fu Z, Zhang R, Cui Y. Understanding the anchoring effect of two-dimensional layered materials for lithium–sulfur batteries. *Nano Lett.* **15**, 3780-3786 (2015).

8. Kwon S, Youn S, Min B. Itinerant ferromagnetism in half-metallic CoS 2. *Phys. Rev. B* **62**, 357 (2000).

9. Wang L, Maxisch T, Ceder G. Oxidation energies of transition metal oxides within the GGA+ U framework. *Phys. Rev. B* **73**, 195107 (2006).

10. Sai Gautam G, Carter EA. Evaluating transition metal oxides within DFT-SCAN and $\text{SCAN+U$ frameworks for solar thermochemical applications. *Phys. Rev. Mater.* **2**, 095401 (2018).

11. Ong SP, *et al.* Python Materials Genomics (pymatgen): A robust, open-source python library for materials analysis. *Comp. Mater. Sci.* **68**, 314-319 (2013).

12. Mathew K, Sundararaman R, Letchworth-Weaver K, Arias T, Hennig RG. Implicit solvation model for density-functional study of nanocrystal surfaces and reaction pathways. *J. chem. phy.* **140**, 084106 (2014).

13. Riadigos C, Iglesias R, Rivas M, Iglesias T. Permittivity and density of the systems (monoglyme, diglyme, triglyme, or tetraglyme+ n-heptane) at several temperatures. *J. Chem. Therm.* **43**, 275-283 (2011).

14. Nowack E, Schwarzenbach D, Hahn T. Charge densities in CoS2 and NiS2 (pyrite structure). *Acta Crystallogr. Sect. B: Struct. Sci.* **47**, 650-659 (1991).

15. Zhang L, *et al.* Self-Assembling Hollow Carbon Nanobeads into Double-Shell Microspheres as a Hierarchical Sulfur Host for Sustainable Room-Temperature Sodium–Sulfur Batteries. *ACS Appl. Mater. Interfaces* **10**, 20422-20428 (2018).

16. Wang C, *et al.* Frogspawn-Coral-Like Hollow Sodium Sulfide Nanostructured Cathode for High-Rate Performance Sodium–Sulfur Batteries. *Adv. Energy Mater.* **9**, 1803251 (2019).

17. Zheng S, Han P, Han Z, Li P, Zhang H, Yang J. Nano-Copper-Assisted Immobilization of Sulfur in High-Surface-Area Mesoporous Carbon Cathodes for Room Temperature Na-S Batteries. *Adv. Energy Mater.* **4**, 1400226 (2014).

18. Xin S, Yin YX, Guo YG, Wan LJ. A high-energy room-temperature sodium-sulfur battery. *Adv. Mater.* **26**, 1261-1265 (2014).

19. Lu X, *et al.* Advanced intermediate-temperature Na–S battery. *Energy & Environ Sci.* **6**, 299-306 (2013).

20. Hwang TH, Jung DS, Kim J-S, Kim BG, Choi JW. One-Dimensional Carbon–Sulfur Composite Fibers for Na–S Rechargeable Batteries Operating at Room Temperature. *Nano Lett.* **13**, 4532-4538 (2013).

21. Wei S, *et al.* A stable room-temperature sodium–sulfur battery. *Nat. Commun.* **7**, 11722 (2016).

22. Zhang B-W, *et al.* Atomic cobalt as an efficient electrocatalyst in sulfur cathodes for superior room-temperature sodium-sulfur batteries. *Nat. Commun.* **9**, 4082 (2018).

23. Yu X, Manthiram A. Performance Enhancement and Mechanistic Studies of Room-Temperature Sodium–Sulfur Batteries with a Carbon-Coated Functional Nafion Separator and a Na2S/Activated Carbon Nanofiber Cathode. *Chem. Mater.* **28**, 896-905 (2016).

24. Yao Y, Zeng L, Hu S, Jiang Y, Yuan B, Yu Y. Binding S0.6Se0.4 in 1D Carbon Nanofiber with C□S Bonding for High-Performance Flexible Li–S Batteries and Na–S Batteries. *Small* **13**, 1603513 (2017).

25. Wang Y-X, *et al.* Achieving High-Performance Room-Temperature Sodium–Sulfur Batteries With S@Interconnected Mesoporous Carbon Hollow Nanospheres. *J. Am. Chem. Soc.* **138**, 16576-16579 (2016).

26. Lu Q, *et al.* Freestanding carbon fiber cloth/sulfur composites for flexible room-temperature sodium-sulfur batteries. *Energy Storage Mater.* **8**, 77-84 (2017).

27. Qiang Z, Chen Y-M, Xia Y, Liang W, Zhu Y, Vogt BD. Ultra-long cycle life, low-cost room temperature sodium-sulfur batteries enabled by highly doped (N,S) nanoporous carbons. *Nano Energy* **32**, 59-66 (2017).
